# Goblet Cell Carcinoids: Characteristics of a Danish Cohort of 83 Patients

**DOI:** 10.1371/journal.pone.0117627

**Published:** 2015-02-11

**Authors:** Ingrid Holst Olsen, Nanna Holt, Seppo W. Langer, Jane P. Hasselby, Henning Grønbæk, Jens Hillingsø, Masti Mahmoud, Morten Ladekarl, Lene H. Iversen, Andreas Kjær, Birgitte H. Federspiel, Ulrich Knigge

**Affiliations:** 1 Department of Surgical Gastroenterology, European NET Centre of Excellence, Rigshospitalet, Faculty of Health Sciences, University of Copenhagen, Copenhagen, Denmark; 2 Department of Oncology, European NET Centre of Excellence, Rigshospitalet, Faculty of Health Sciences, University of Copenhagen, Copenhagen, Denmark; 3 Department of Pathology, European NET Centre of Excellence, Rigshospitalet, Faculty of Health Sciences, University of Copenhagen, Copenhagen, Denmark; 4 Department of Endocrinology, European NET Centre of Excellence, Rigshospitalet, Faculty of Health Sciences, University of Copenhagen, Copenhagen, Denmark; 5 Department of Clinical Physiology, Nuclear Medicine and PET and Cluster for Molecular Imaging, European NET Centre of Excellence, Rigshospitalet, Faculty of Health Sciences, University of Copenhagen, Copenhagen, Denmark; 6 Department of Hepatology and Gastroenterology, European NET Centre of Excellence, Aarhus University Hospital, Aarhus, Denmark; 7 Department of Oncology, European NET Centre of Excellence, Aarhus University Hospital, Aarhus, Denmark; 8 Department of Surgery, European NET Centre of Excellence, Aarhus University Hospital, Aarhus, Denmark; University of Verona, ITALY

## Abstract

**Background:**

Appendiceal goblet cell carcinoids (GCCs) exhibit neuroendocrine and adenocarcinoma features.

**Patients and Methods:**

Analysis of demography, pathology, prognostic markers, treatment and survival in 83 GCC patients (f/m: 56/27) diagnosed 1992-2013.

**Results:**

Median age for f/m was 59/58 years, respectively, and similar for localized and disseminated disease. At diagnosis 54 patients had localized appendiceal disease (f/m: 29/25). According to TNM 24% had Stage I, 70% had Stage II and 6% had Stage III. Twenty-nine patients had disseminated disease (f/m: 27/2). Chromogranin A, synaptophysin and p53 were positive in >90%. Serotonin was positive in 70%. Median Ki67 index was 32% (6-75%) and higher in Tang group C (50%) compared to group A (30%; p<0.0001), and group B (30%; p<0.004). All patients had surgery. Sixty-three (76%) had radical resections including all patients with localized disease. Median OS was 83 months. The 1-, 5- and 10-year survival rates were 90%, 58%, and 38%, respectively. For localized disease OS was 164 months and 1-, 5- and 10-year survival rates were 100%, 80%, and 55%, respectively. For disseminated disease OS was 19 months and 1-, 5- and 10-year survival rates were 73%, 18% and 6%, respectively. The 1-, 5- and 10 year-survival rates for f/m were 87%/96%, 49%/76% and 31%/57%, respectively (p = 0.02). According to the Tang classification group A, B, and C OS was 118, 83 and 20 months, respectively (p = 0.0002).

**Conclusion:**

The Tang classification was found to be a significant prognostic factor, while the Ki67 index was not. Localized GCCs occurred equally in males and females, while disseminated GCCs were mostly seen in females. Median age of patients with localized disease and disseminated disease was identical. Cox regression analysis found Stage IV, focally positive synaptophysin and non-radical surgery as strongest negative prognostic factors.

## Introduction

Goblet cell carcinoids/carcinomas (GCCs) are considered a subgroup of mixed neuroendocrine neoplasms (NENs) and adenocarcinomas occurring with an incidence of approximately 0.01–0.05/100,000/year and occur almost exclusively in the appendix [1–3]. GCCs were first described as a separate entity from adenocarcinomas and carcinoid tumors in 1974 [4]. GCCs are more aggressive than typical appendiceal carcinoid tumors but less aggressive than adenocarcinomas [5–8].

According to the SEER database of 227 GCCs registered in 1973–1998, the mean age at diagnosis was 52 years with a second peak in the seventies [2]. Thus, patients with GCCs are approximately 1–3 decades older than patients with appendiceal NENs [1,6,7,9]. A higher incidence among Caucasians is described [2,10].

According to the SEER database the female-male ratio in GCC patients is equal [2]. However, smaller case-series (< 65 patients) report either an equal gender distribution [7,11] or an overweight of female patients [8,9]. In The World Health Organization (WHO) tumor classification from 2010 [3] GCC is considered a subgroup of mixed adenoneuroendocrine carcinomas (MANECs) and is in the Tumor-Nodes-Metastasis (TNM) classification of malignant tumors (Union for International Cancer Control (UICC) / American Joint Committee on Cancer (AJCC) / European Neureoendocrine Tumor Society (ENETS)) classified according to the scheme for adenocarcinoma [1,12,13].

Tang et al. proposed a pathological subclassification of GCCs based only on the morphology of the primary tumor [9]. This subclassification (Tang group A, B and C) has proved useful for predicting clinical behavior and prognosis.

Approximately 50–60% of the patients presented with symptoms of acute appendicitis [1]. However, one third of the patients were asymptomatic and the GCC was identified incidentally after appendectomy performed in addition to other surgery [7,14,15]. Other patients presented with chronic intermittent abdominal pain, palpable abdominal mass, gastrointestinal bleeding and weight loss. Less than 1% of patients had an established preoperative diagnosis of a primary appendiceal GCC [1,9,16]. At diagnosis, approximately 10% of GCCs were disseminated with distant metastases to the ovaries, the peritoneum, distant lymph nodes, liver, and bones [1]. Females with ovarian masses were generally presumed preoperatively to have a primary ovarian cancer [9].

The aim of this retrospective study was to characterize a large cohort of Danish patients with appendiceal GCCs obtained from the databases at the ENETS Centers of Excellence at Rigshospitalet, Copenhagen University Hospital and at Aarhus University Hospital, Denmark.

## Material and Methods

### Patient identification

From the NEN-databases 83 patients with primary appendiceal GCC were in the period May 1992 to April 2013 identified at Rigshospitalet, Copenhagen University Hospital (n = 59) and at Aarhus University Hospital (n = 24), Denmark. Patients had regular follow-up every 3–12 months dependent on state of disease. Given the retrospective nature of the study, the follow-up procedures varied in terms of time intervals and available clinical procedures (biochemical markers and imaging). Patients were followed until the 1^st^ of August 2014 or death, whichever came first. No patients were lost to follow-up and the median follow-up time was 48 (range 2–241) months. The Danish National Committee on Health Research Ethics approved the study (trial registration ID: H-D-2007–0083). As a retrospective database study the National Committee on Health Research Ethics did not require written or verbal informed consent. The NEN-databases were approved by The Danish Data Protection Agency.

Demographic data and clinical variables were recorded and presented in Tables 1, 2 and 3. Radical surgery refers to R0 or R1 and non-radical surgery to R2 [17].

Localized disease was defined as GCC restricted to the appendix and regional lymph nodes (Stage I-III) and disseminated disease as metastases e.g. to the ovaries, liver or peritoneum (Stage IVA and IVB). Stage grouping was based on pathological (p)TNM and clinical (c)TNM, whenever possible (n = 77) [13].

**Table 1:** Patient Characteristics.

	ALL		LOCALIZED AT DIAGNOSIS		DISSEMINATED AT DIAGNOSIS	
	n	Percent (%)	n	Percent (%)	n	Percent (%)
No of Patients	83		54	65	29	35
Copenhagen	59	71	37	69	22	76
Aarhus	24	29	17	31	7	24
Diagnosed 1992–2002	17	20	9	53	8	47
Diagnosed 2003–2013	66	80	45	68	21	32
Female:Male	56:27		29:25		27:2	
Median Age at Diagnosis, years, (Range)	59 (31–77)		59 (31–77)		58 (41–76)	
Female (n = 56)	59 (31–76)		60 (31–76)		57 (41–71)	
Male (n = 27)	58 (33–77)		57 (33–77)		68 (59–76)	
						
Primary Symptom						
Appendicitis	48	58	45	83	3	10
Abdominal pain	9	11	5	9	4	14
Symptoms of c.ovarii	18	22	1	2	17	59
Carcinoid Syndrome	1	1	0	0	1	3
Others	7	8	3	6	4	14
Tumor Stage Grouping	77	93	50		27	
I	12	16	12	24	0	0
II	35	45	35	70	0	0
III	3	4	3	6	0	0
IVA	3	4	0	0	3	11
IVB	24	31	0	0	24	89
Immunohistochemistry (IHC)						
Chromogranin A						
Positive	71	86	48	89	23	79
Focally positive	12	14	6	11	6	21
Negative	0	0	0	0	0	0
Synaptophysin						
Positive	73	88	49	91	24	83
Focally positive	9	11	5	9	4	14
Negative	1	1	0	0	1	3
Serotonin						
Positive	25	30	16	30	9	31
Focally positive	32	39	22	40	10	34,5
Negative	25	30	15	28	10	34,5
Not performed	1	1	1	2	0	0
p53						
Positive	17	21	14	26	3	10
Focally positive	56	67	37	69	19	66
Negative	4	5	0	0	4	14
Not performed	6	7	3	5	3	10
MUC1/EMA						
Positive	66	80	44	81	22	76
Focally positive	2	2	1	2	1	3
Negative	7	8	2	4	5	17
Not performed	8	10	7	13	1	3
MUC2						
Positive	73	88	47	87	26	90
Focally positive	1	1	0	0	1	3
Negative	4	5	2	4	2	7
Not performed	5	6	5	9	0	0
Survivin (positivity of nucleus)						
Median, % (range)	15 (0–50)		15 (0–50)		15 (3–50)	
						
Ki-67						
Median (%), (range)	32 (6–75)		30 (6–75)		35 (10–65)	
≤20%	14	17	9	17	5	17
>20%	69	83	45	83	24	83
TANG Classification						
A	34	41	30	55	4	14
B	40	48	23	43	17	59
C	9	11	1	2	8	27

**Table 2:** Immunohistochemistry (IHC).

(n (%))	Ki67 index		Tang Classification*		
	≤20% (n = 14)	> 20% (n = 69)	A (n = 34)	B (n = 40)	C (n = 9)
Tumor Stage Grouping (n = 77)	14	63	30	38	9
I	2 (14%)	10 (16%)	8 (27%)	4 (11%)	0
II	6 (43%)	29 (46%)	18 (60%)	16 (42%)	1 (11%)
III	1 (7%)	2 (3%)	1 (3%)	2 (5%)	0
IVA	1 (7%)	2 (3%)	0	3 (8%)	0
IVB	4 (29%)	20 (32%)	3 (10%)	13 (34%)	8 (89%)
CgA					
Positive	12(86%)	59 (86%)	31 (91%)	34 (85%)	6 (67%)
Focally Positive	2 (14%)	10 (14%)	3 (9%)	6 (15%)	3 (33%)
Synaptophysin					
Positive	12 (86%)	61 (88%)	33 (97%)	34 (85%)	6 (67%)
Focally Positive	1 (7%)	8 (12%)	1 (3%)	5 (13%)	3 (33%)
Negative	1 (7%)	0	0	1 (2%)	0
Serotonin					
Positive	4 (28%)	21 (31%)	11 (32%)	14 (35%)	0
Focally Positive	5 (36%)	27 (39%)	16 (47%)	11 (27%)	5 (56%)
Negative	5 (36%)	20 (29%)	6 (18%)	15 (38%)	4 (44%)
Not Performed	0	1 (1%)	1 (3%)	0	0
p53					
Positive	3 (22%)	14 (20%)	3 (9%)	11 (27%)	3 (33%)
Focally Positive	9 (64%)	47 (68%)	28 (82%)	24 (60%)	4 (44%)
Negative	0	4 (6%)	1 (3%)	2 (5%)	1 (11%)
Not Performed	2 (14%)	4 (6%)	2 (6%)	3 (8%)	1 (11%)
MUC1					
Positive	9 (65%)	57 (83%)	29 (85%)	30 (74%)	7 (78%)
Focally Positive	1 (7%)	1 (1%)	2 (6%)	0	0
Negative	2 (14%)	5 (7%)	0	5 (13%)	2 (22%)
Not Performed	2 (14%)	6(9%)	3 (9%)	5 (13%)	0
MUC2					
Positive	13 (93%)	60 (87%)	31 (91%)	35 (87%)	7 (78%)
Focally Positive	0	1 (1%)	0	0	1 (11%)
Negative	0	4 (6%)	0	3 (8%)	1 (11%)
Not Performed	1 (7%)	4 (6%)	3 (9%)	2 (5%)	0
Survivin (% positivity of nucleus), median (range)	9 (3–18)	16 (0–50)	15 (1–40)	15 (0–50)	20 (10–50)
Ki67 Index (%), median (range)	15 (6–20)	35 (22–75)	30 (6–50)	30 (10–75)	50 (30–60)

*Tang LH et al. Pathologic Classification and Clinical Behavior of the Spectrum of Goblet Cell Carcinoid Tumors of the Appendix. The American Journal of Surgical Pathology. 2008;32:1429–43.

**Table 3:** Treatment and Imaging.

	ALL		LOC. AT DIAGNOSIS		DISS. AT DIAGNOSIS	
	n	Percent (%)	n	Percent (%)	n	Percent (%)
Treatment (all patients had primary tumor resected)	83		54	65	29	35
Surgical Intervention						
Appendectomy alone	4	5	3	6	1	4
Appendectomy + Right-sided hemicolectomy (RHC)	53	64	48	89	5	17
Appendectomy+RHC+ bilateral salpingo-oophorectomy (BSO)/hysterectomy	16	29	3	10	13	48
Appendectomy+ BSO/hysterectomy (No RHC performed)	10	18	0	0	10	37
Cytoreductive surgery and HIPEC	4		1	25	3	75
Radical resection (R0/R1)	63	76	54	100	9	31
Non-radical resection (R2)	20	24	0	0	20	69
Medical treatment (chemotherapy)	24	29	6*a	25	18	75
Small-cell-lung-cancer (SCLC) regimen*1	11	13	4	7	7	24
Colorectal cancer (CRC) regimen*2	9	11	2	4	7	24
Neuroendocrine Tumor regimen*3	3	4	0	0	3	10
Ovarian cancer regimen*4	1	1	0	0	1	4
No chemotherapy	59	71	48	89	11	38
Imaging						
Somatostatin Receptor Scintigraphy (SRS), post operative						
Performed	48	58	31		17	
Not Performed	35	42	23		12	
Positive	5	10	1	3	4	24
Negative	43	90	30	97	13	76

*1: carboplatin+etoposide (n = 10); cisplatin+topotecan (n = 1).

*2: capecitabine+oxaliplatin (n = 6); irinotecan (n = 1); FOLFOX (n = 1); bevazicumab+oxaliplatin+capecitabine (n = 1).

*3: streptozocin+5-FU (n = 3).

*4: carboplatin+docetaxel (n = 1).

*a: After relapse.

Abbreviations: LOC (Localized), DISS. (Disseminated).

### Morphology and Immunohistochemistry

**Fig 1:**
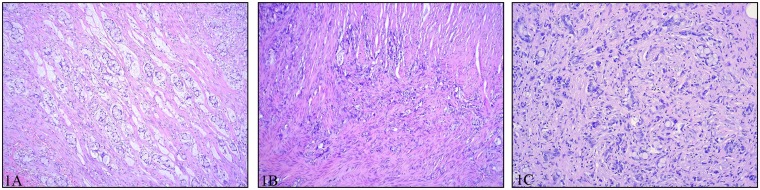
1A. Tang group A. Typical GCC with goblet cell groups and strands of goblet cells separating muscle fibers. No desmoplasia. **1B. Tang group B.** Irregular groups of signet ring like cells with nuclear pleomorphism are seen in the muscularis propria. Only few groups of typical goblet cells (not shown in this photo) were found. **1C. Tang group C.** Areas of tumor indistinguishable from a poorly differentiated adenocarcinoma are present. In this field a few small groups of typical goblet cells are seen revealing the tumor’s true nature.

Tissue from the primary appendiceal GCC was available from all 83 patients. Hematoxylin and eosin stained sections were reviewed to confirm the diagnosis and to apply the Tang Classification (Fig. 1A, 1B, 1C) [9]. When possible the pTNM and Stage (S1 Table) were determined according to UICC [13] (Table 1).

All 83 tumors were immunostained using the antibodies listed in S2 Table, where more details of the immunoreactions are depicted.

Ki67 and survivin immunoreactivity were expressed as the mean percentage of tumor cells with the highest nuclear labeling after counting 20 hot spot areas. Based on the Ki67 index the tumors were classified into subgroups: Ki67 ≤2%, Ki67 3–20% and Ki67 >20% as used for NENs according to the WHO 2010 Classification [3].

For the remaining immunostains, immunoreactivity was scored as positive (more than 30% of the tumor cells reacted), focally positive (1–30% of the tumor cells reacted) or negative (less than 1% of the tumor cells reacted). Two pathologists reviewed the slides first separately and when there was disagreement, the cases were discussed at the double-headed microscope until agreement was reached.

### Statistical analysis

Data are presented as median and range. Descriptive statistics were used to characterize clinical parameters. One-way analysis of variance was used for comparison of continuous variables between groups. The association of categorical variables was assessed by chi-square test or Fisher’s exact test when appropriate and the t-test, Mann-Whitney test for continuous variables. Overall survival (OS) was defined as the time from the date of diagnosis to the date of death from any cause or last follow-up. Relapse free survival (RFS) was defined as time form primary radical treatment to development of first evidence of clinical or radiological proven metastatic disease. OS and RFS were estimated by the Kaplan-Meier method, and significance was tested by the log-rank test using the IBM SPSS Statistics 20 software (IBM, Armonk, USA). A p-value below 0.05 was considered statistically significant.

Multivariate analyses using the Cox proportional hazards regression model were performed to identify factors independently associated with prognosis. Covariates in the univariate analysis are given in Table 4. All significant factors from the univariate analysis were included in the multivariate analyses (Table 4). The receiver operating characteristic (ROC) method was used to identify the value of survivin and Ki67 index (continuous variables) as prognostic factors and if applicable the best cut-offs were used. No cut-off for survivin could be determined by the ROC method; hence the median value of 15 was used to subdivide into two groups used in the univariate analyses.

**Table 4:** Cox Regression on Prognostic Baseline Factors for Survival in patients with GCC (n = 83).

Variables	HR (95% CI)	p value	HR (95% CI)	p value
	Univariate analysis		Multivariate analysis	
Gender Female (vs. Male)	2.60 (1.1–5.8)	0.022		
Age (y)	1.0 (1.0–1.1)	0.346		
Disseminated Disease (vs. Localized Disease)	7.8 (4.0–15.1)	<0.0001		
Non-radical Surgery (vs. Radical Surgery)	6.7 (3.5–12.7)	<0.0001	3.2 (1.2–8.5)	0.023
CgA Focally Positive (vs. Positive CgA)	2.2 (1.0–4.6)	0.044		
Synaptophysin*	2.7 (1.2–6.2)	0.021	2.6 (1.0–6.7)	0.042
Serotonin*	1.5 (1.0–2.2)	0.050		
p53*	2.2 (1.1–4.6)	0.031		
MUC1*	1.2 (0.7–1.9)	0.580		
MUC2*	1.3 (0.7–2.5)	0.409		
Survivin (≤14% vs. >14%)	1.6 (0.8–3.3)	0.136		
Ki67 (<20% vs. >20%)	0.7 (0.3–1.3)	0.178		
Stage Grouping (I vs. II vs. III vs. IVA vs. IVB)	2.1 (1.6–2.7)	<0.0001	2.5 (1.6–3.8)	<0.0001
Tang Classification (A vs. B vs. C)	2.7 (1.6–4.5)	<0.001		

* positive vs. *focally positive vs*.*negative*.

## Results

### Patient characteristics

Patient characteristics are given in Table 1. Of the 83 patients, 54 patients (65%) had localized disease and 29 patients (35%) had disseminated disease at diagnosis.

The cohort consisted of 67% females and 33% males. In patients with localized disease the female/male ratio was 29/25. Disseminated disease was found in 48% (n = 27) of the females and in 7% (n = 2) of the males (p<0.0001).

The median age at diagnosis was 59 years (range 31–77). There was no difference in age between the subgroups localized disease vs. disseminated disease at diagnosis (59 vs. 58 years; p = 0.81) or gender in the entire cohort (female vs. male; 59 vs. 58 years; p = 0.82) or in the subgroups localized females vs. localized males (60 vs. 57 years; p = 0.63) and disseminated females vs. disseminated males (57 vs. 68 years; p = 0.10). Median age for patients diagnosed as Tang group A, B or C was 59 years (range 31–77), 59 years (range 42–76) and 49 years (range 42–60), respectively (p = 0.18).

### Clinical presentation

The most common clinical presentation was symptoms of acute appendicitis (58%) followed by symptoms similar to ovarian cancer (Table 1). One patient presented with the carcinoid syndrome. In patients with localized disease 85% (n = 45) presented with symptoms of appendicitis whereas 59% (n = 17) of patients with disseminated disease presented with symptoms suspicious for ovarian cancer.

### Tumor Stage Grouping

Determination of TNM and Stage grouping was possible in 77 (93%) patients (Table 1 and Table 2). Fifty patients were classified as Stage I-III and 27 patients were classified as Stage IV. Of these 89% had distant metastases in more than one organ.

TNM and Stage grouping are specified in S1 Table. No correlation was found between Ki67 index and Stage grouping (Table 2). In Tang group A, 27 of the patients had tumor Stage I, II or III (Table 2). The three patients having Stage IVB in Tang group A were all females, two patients had non-radical surgery and one had radical resection but relapsed subsequently. In Tang group B 22 patients had Stage I-III and 16 Stage IV. In Tang group C one patient had Stage II and 8 patients had Stage IV.

### Clinical management

**Surgical treatment**

All 83 patients had resection of the primary tumor (Table 3) of which 76% had a radical resection. Of the 54 patients with localized disease 94% had an appendectomy followed by a right-sided hemicolectomy (RSH) and 6% (n = 4) had a simple appendectomy. These four patients all belonged to Tang group A of which three are still alive. All 54 patients had radical resections. Three patients had a bilateral salpingo-oophorectomy (BSO) more than 1 year later due to development of ovarian metastases.

Of the 29 patients with disseminated disease at diagnosis, only 9 (31%) had radical resections (Table 3).

Four patients received hyperthermic intraperitoneal chemotherapy (HIPEC) with mitomycin C in relation to surgery (one male and three females), of whom three presented with disseminated disease at diagnosis and one with a perforated appendicitis. One patient was classified as Tang group A, the remaining three as Tang group B.

**Chemotherapy**

Of the 83 patients 29% (n = 24) received chemotherapy (Table 3). None of the 54 patients with localized disease had adjuvant therapy after primary surgery, however, six of these had chemotherapy after relapse.

Of the 29 patients with disseminated disease at initial presentation 11 (38%) did not receive systemic chemotherapy, while 18 (62%) of which 13 (72%) had non-radical resections, received systemic chemotherapy. First-line regimens used are shown in Table 3.

### Morphology and immunohistochemistry (IHC)

Histological re-evaluation confirmed the diagnosis of GCC in all 83 patients (Table 1). There was no significant difference in positivity of CgA, synaptophysin and serotonin between localized and disseminated disease.

The tumor suppressor protein p53, determined in 93% of the patients, was positive or focally positive in all patients with localized disease (n = 51) vs. 76% (n = 22) of patients with disseminated disease at diagnosis (p = 0.004). There was no difference in gender regarding neuroendocrine markers and p53.

MUC1 and MUC2 were positive in the majority of the patients independent of tumor spread and survivin showed no significant difference between localized vs. disseminated disease (Table 1).

The median Ki-67 proliferation index was 32% and almost identical in patients with localized or disseminated disease (p = 0.62;Table 1). In both groups 17% had a Ki-67 index ≤ 20% and 87% had a Ki-67 index >20% (Table 1).

According to the Tang classification 41%, 48% and 11% belonged to group A, B and C, respectively (Table 1). When comparing localized vs. disseminated disease, 55% vs. 14% belonged to group A (p<0.001), 43% vs. 59% to group B (p = 0.11) and 2% vs. 27% to group C (p<0.001) (Table 1).

Table 2 reflects results for the individual IHC markers to the Ki67 index and the Tang A, B and C Classification, respectively.

The neuroendocrine markers CgA and synaptophysin were positive in 85% or more in Tang group A and B decreasing to 67% in Tang group C (CgA: Tang group A vs. B p = 0.11; Tang group A vs. C p = 0.002 and Tang group B vs. C p = 0.038; synaptophysin: Tang group A vs. B p = 0.01; Tang group A vs. C p<0.0001 and Tang group B vs. C p = 0.016). Serotonin staining was positive or focally positive in 79% and 62% in Tang group A and B, respectively, but only focally positive in 56% in Tang group C (Tang group A vs. B p<0.001; Tang group A vs. C p = 0.025 and Tang group B vs. C p = 0.555).

Almost identical percentages of median Ki67 index were found for localized and disseminated disease, respectively (30% vs. 34%; p = 0.66; Table 2).

Median Ki67 index was significantly higher in Tang group C (50%) compared to Tang group A (30%; p<0.0001), and Tang group B (30%; p< 0.004) (Table 2).

Survivin was positive in 9% of patients with a Ki67 ≤ 20% compared to 16% with a Ki67 > 20% (p<0.0001; Table 2), however, there was no significant difference in survivin positivity in the three Tang groups. Survivin and Ki67 were tested as prognostic markers by ROC curve analysis with AUCs of 0.55 (95% CI: 0.42–0.68, SE = 0.07) for survivin and of 0.46 (95% CI: 0.33–0.58, SE = 0.07) for Ki67, respectively. However, as continuous variables neither survivin nor Ki67 were useful as prognostic markers for GCCs. Furthermore, the Ki67 index as a categorical variable (Ki67 <20% vs. Ki67 >20%) was insignificant in our univariate analysis (Table 4).

### Postoperative Somatostatin Receptor Imaging

Somatostatin receptor scintigraphy (SRS) was performed post-operatively in 48 patients (58%) (Table 3). Of these 13 patients (27%) had residual tumor and SRS was positive in 5 (38%). Of patients with disseminated disease 17 had a SRS of which 4 (24%) had a positive uptake in the residual tumor. SRS was performed in 31 patients with localized disease of which one was positive (3%). No patients had false positive SRS. Thirty-five patients (73%) had no residual tumor and negative SRS.

### Survival and prognostic factors

**Fig 2:**
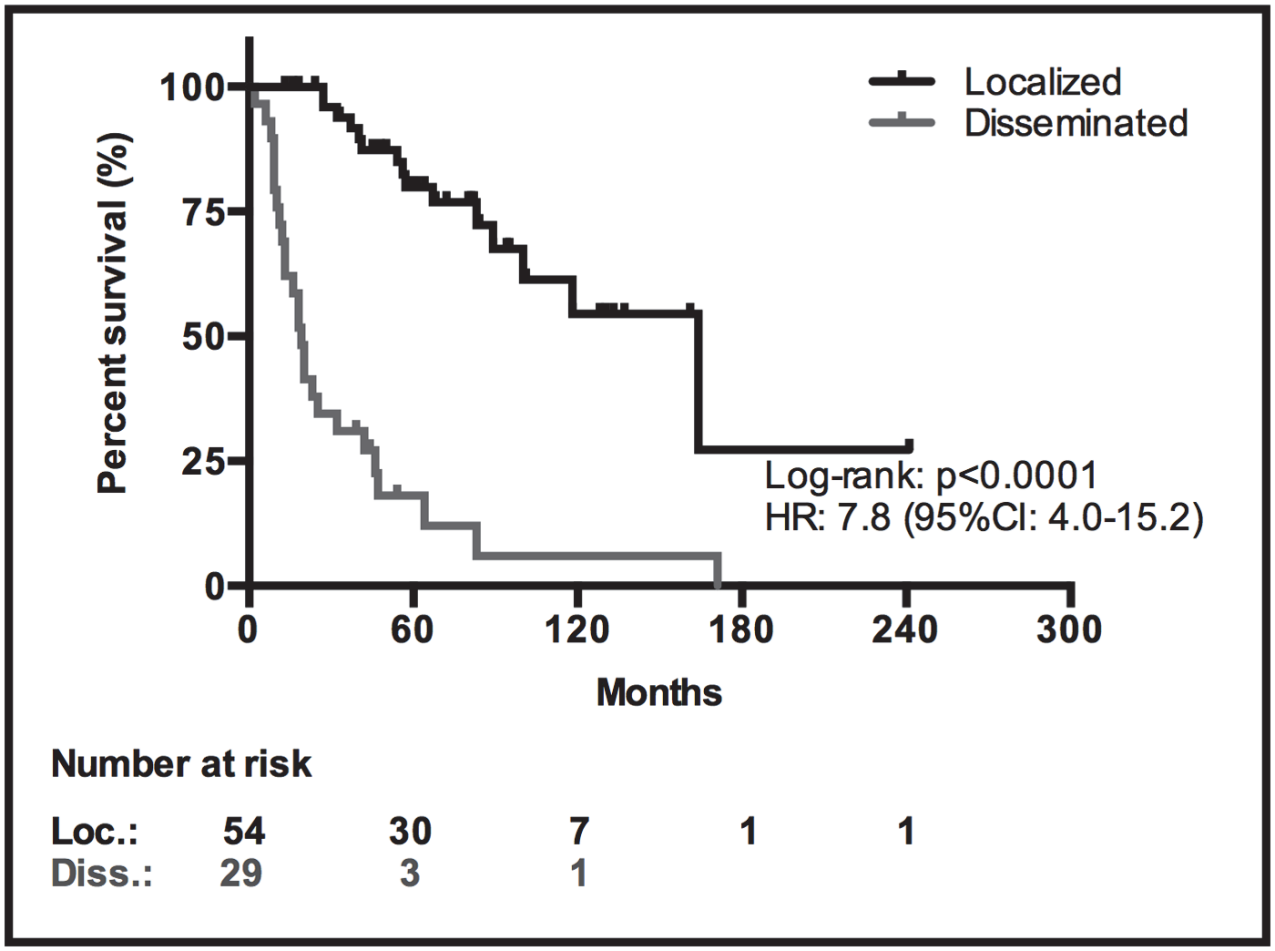
Kaplan-Meier survival curves for localized vs. disseminated goblet cell carcinoid at diagnosis.

The median OS of the 83 patients was 83 months (95% CI: 51–115 months), with 42 patients (51%) being alive at last follow-up. The one-, five- and ten-year survival rates were 90%, 58% and 38%, respectively. The median OS for the 54 patients with localized disease at diagnosis was 164 months (95% CI: 90–238 months). The one-, five- and ten-year survival rates were 100%, 80% and 55%, respectively. In the 29 patients with disseminated disease the median OS was 19 months (95% CI: 15–23 months). The one-, five- and ten-year survival rates were 73%, 18% and 6%, respectively. The difference in survival between the two groups was highly significant (HR: 7.8, 95% CI: 4.0–15.2; p<0.0001) (Fig. 2).

Eighteen patients (29%) of the radically resected subgroup had relapse. Time from primary radical resection to development of first evidence of clinical or radiological proven metastatic disease, determined as relapse free survival (RFS), was 29 months (95% CI: 17–41). For this group OS from diagnosis was 42 months (95% CI: 30–54).

Seven patients with disseminated disease at diagnosis, subsequently radical operated had metastatic relapse within 16 months (95% CI: 8.3–23.7 months) vs. the subgroup of patients with localized disease (n = 11) that had a RFS of 33 months (95% CI: 26.5–39.5) (HR 2.8, 95% CI: 1.0–8.2; p = 0.048).

Patients presenting with symptoms of acute appendicitis (n = 48; 58%) survived significantly longer (median not reached) vs. the subgroup of patients presenting with other symptoms (n = 35, 42%) listed in Table 1 (27 months) (HR: 1.2, 95% CI: 1.1–1.3; p<0.0001).

**Fig 3:**
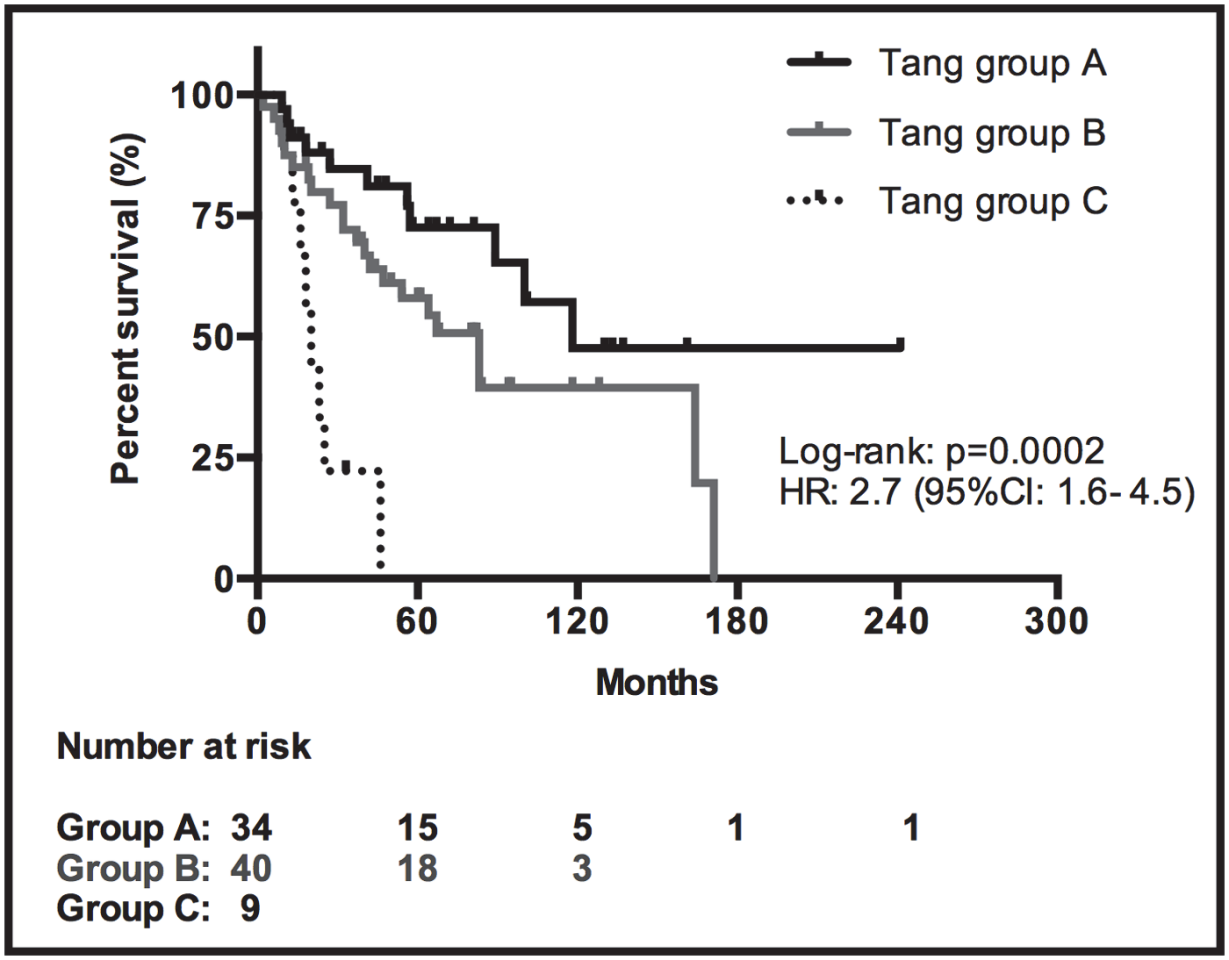
Kaplan-Meier survival curves of the Tang classification.

The Tang classification correlated with survival. The median OS was 118 months for Tang group A (SE/CI95% not calculated, since median just reached), 83 months (95% CI: 53–113) for Tang group B and 20 months (95% CI: 14–26) for Tang group C. A Kaplan-Meier curve and log-rank overall comparison, demonstrated a highly significant difference (HR: 2.7, 95% CI: 1.6–4.5; p = 0.0002) (Fig. 3). Log-rank pairwise comparisons identified significant differences between Tang group A and C (HR: 3.0, 95% CI: 1.7–5.1; p = 0.0001), between Tang group B and C (HR: 4.3, 95% CI: 1.7–10.6; p = 0.002) and a non-significant difference in OS between Tang group A and B (HR: 1.9, 95% CI: 0.9–4.0; p = 0.08).

There was no significant difference in OS between patients with Ki67 index <20% (57 months (95% CI: 21–93)) and patients with Ki67 index above 20% (89 months (95% CI: 35–143)), p = 0.25.

The median OS for patients with disseminated disease receiving chemotherapy was 20 months (95% CI: 9.6–30.4) vs. 18 months (95% CI: 0.5–2.9) for patients with disseminated disease not receiving chemotherapy (HR 1.2; p = 0.63). The median OS for patients receiving chemotherapeutic regimens as for CRC (colorectal-cancer) cancer (n = 9) was 42 months (95% CI: 13–71 months) and for patients having SCLC (small-cell-lung-cancer)-regimens (n = 11) 23 months (95% CI: 13–33 months) (p = 0.42).

**Fig 4:**
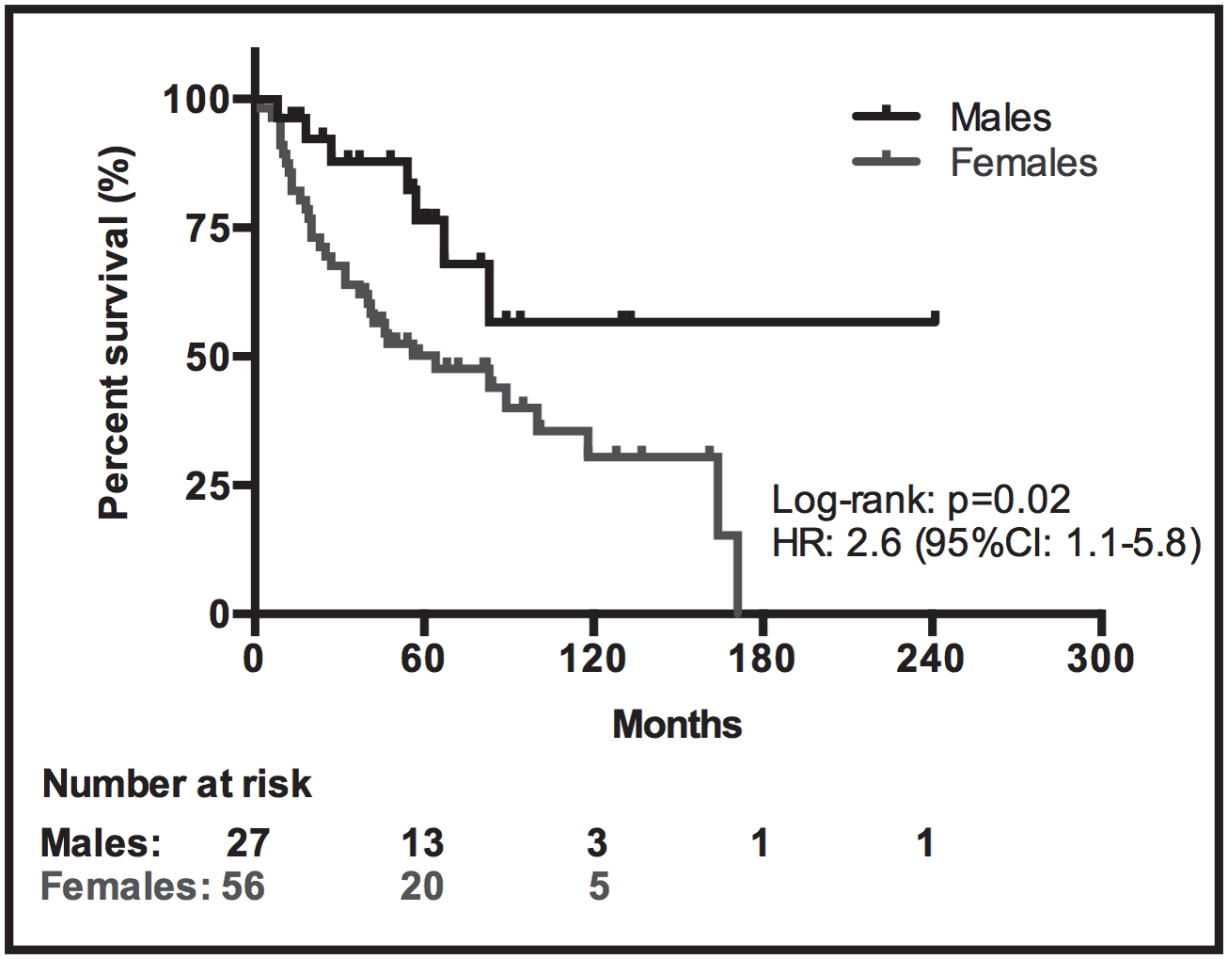
Kaplan-Meier survival curves for males vs. females.

At last follow-up, August 2014, 20 of 27 (74%) males and 22 of 56 (39%) females were still alive. The median OS for females was 64 months (95% CI: 19–109 months) and for males the median was not reached (HR: 2.6, 95% CI: 1.1–5.8; p = 0.02;Fig. 4).Fig. 4 may be biased since more females had disseminated disease than males in our cohort. There was no difference in OS between males and females in the subgroup with disseminated disease (8 months vs. 20 months (p = 0.13)) or in the subgroup with localized disease (p = 0.61), 80% of males (20/25) and 66% (19/29) of females being alive at last follow-up. Females with disseminated disease had a significantly shorter survival (20 months) compared to females with localized disease at diagnosis (164 months) (HR: 5.9, 95% CI: 2.7–12.9; p<0.0001).

To perform Cox multivariate analysis, 8 covariates were identified by univariate analysis of all independent factors associated with prognosis (Table 4). As covariates in the Cox analysis gender, localized vs. disseminated disease at diagnosis, Stage, Tang classification, focally positive vs. positive IHC for CgA, negative vs. focally positive vs. positive IHC for synaptophysin and p53 and radical vs. non-radical surgery were included, since all factors were significant in the univariate analysis on prognosis. From the Cox multivariate analysis we concluded that the most negative prognostic factors were non-radical operation and tumor Stage IV. Furthermore, negative and focally positive IHC for synaptophysin were negative prognostic factors compared to positive staining (Table 4).

## Discussion

The present study is one of the largest well characterized cohorts of GCC patients ever reported. The two ENETS Centres of Excellence cover around 75% of the Danish population. The novel findings of the present study were focally positive vs. positive IHC for both CgA and synaptophysin in GCCs, to have Stage IV GCC and non-radical surgery are interpreted as negative prognostic factors. We confirmed that the Tang classification was a significant prognostic factor while the Ki67 index was not associated with overall survival, but Ki67 index correlated with the Tang classification.

The two subgroups, localized and disseminated disease, have similar median age (59 vs. 58 years) and there was no difference in median age between Tang group A, B and C. This may suggest that disseminated disease and morphology according to Tang group C is unrelated to tumor progression over time from localized GCC or Tang group A/B, but may suggest a difference in tumor behavior and biology. We found a 1:1 female:male ratio in the subgroups of localized disease vs. 14:1 in disseminated disease. In previous studies the differences in age or gender were not correlated to localized or disseminated disease [8,9,18,19]. It is unknown why females are more severely affected by disseminated disease than men. All patients in our study had symptoms at diagnosis with 60% showing signs of acute appendicitis as demonstrated by others [1,7,8,20].

The Ki67 proliferation index is mandatory for tumor grading and the malignant potential of gastroenteropancreatic NENs [1,3]. However, the role of Ki67 index in GCCs remains controversial. A retrospective study of 63 GCC patients showed a significantly reduced survival rate with increasing Ki67 index [9]. A recent study of 12 GCCs concluded no prognostic significance of Ki67 for GCCs [19]. In our study median Ki67 was similar for localized and disseminated disease, and the Ki67 index was not a prognostic marker in the univariate or multivariate analysis with similar survival rates in patients with Ki67 below or above 20%. However, patients with Tang group C had a median Ki67 significantly higher than patients with Tang group A and B. Based on our results the Tang grading is more indicative of the behavior of subtypes of GCCs than the Ki67 index. Therefore, careful evaluation of the morphologic features of GCCs is crucial for both clinical management and prediction of outcome and additional IHC markers are warranted.

The oncogene survivin (BIRC5) may be a prognostic marker in gastroenteropancreatic neoplasms and a novel marker for NENs [21,22]. Additionally, adjuvant survivin-targeted therapy may have potential benefit in patients with neuroendocrine carcinomas in the uterine cervix [23]. In the present study we investigated the nuclear expression of survivin, to identify those cancers that were more resistant to apoptotic stimuli and chemotherapy and with poorer survival. However, we found no association between survivin and survival.

Histopathological characterization of GCCs includes positive IHC staining of the NEN markers synaptophysin and CgA [7,9,18,24]. In contrast to typical appendiceal carcinoids which stain more homogenously positive for CgA and synaptophysin, GCCs often show a more scattered positivity of the neuroendocrine markers [1,9,18,25]. Our immunoprofiles of the NENs divided into Tang group A, B and C, are similar to the results by Tang et al. [9]. To complement our univariate analysis we identified both focally positive CgA and focally positive synaptophysin to be independent negative prognostic factors compared to positive staining. This is supported by data regarding the Tang group C that express positive/less positive neuroendocrine immunomarkers—thus resembling poorly differentiated adenocarcinomas [9].

The difference in staining pattern for p53: positive/focally positive vs. negative is in discrepancy with the suggestion of a p53 independent pathway in GCCs [24]. All specimens from localized GCCs expressed positive/focally positive p53. This pattern was not applicable for disseminated GCC and supports our theory of GCCs being a heterogeneous group of diseases. Studies suggest that p53 mutations appear to play a role in the pathogenesis of some GCCs and it is reported that p53 mutation does not necessarily lead to protein overexpression [18,25,26]. In the present study positive p53 expression was determined in both localized and disseminated GCCs and indicates that several p53 dependent pathways are activated in GCCs.

Overexpression of MUC1 has been associated with more aggressive GCC tumor biology [9]. However, we did not find such a correlation. Expression of MUC2, which is secreted from goblet cells in the gut, has been associated with relatively indolent tumor growth [9]. Hence, taking the morphologic Tang classification into consideration the expression of MUC2 should diminish from group A to C. This study does not support MUC1 and MUC2 staining to be a useful tool in classifying GCCs. The discrepancy regarding MUC1 and MUC2 staining in our study compared to Tang et al. (9) may partly be explained by the different antibodies used.

Computer tomography (CT) or magnetic resonance imaging (MRI) is recommended to identify loco-regional or distant metastases [1,27]. Since the presence of somatostatin-receptors on goblet cells is sparse or lacking, SRS is usually not applicable [7,20]. A sensitivity of 38% (5/13) does not justify this imaging procedure in GCCs patients, however, the post-operative SRS performed in our study showed a specificity of 100%. To date there are no specific prospective imaging studies of GCCs. A recent retrospective study of CT demonstrated close correlation between predefined CT pattern and the Tang classification [9,28]. ^18^F-Fluorodeoxyglucose positron emission tomography (FDG-PET) may be useful in patients with increased metabolic activity and high Ki67 index [29].

Treatment of GCCs is primarily based on surgery. RSH is considered the standard surgical treatment of localized GCCs and is recommended to take place within 3 months of the appendectomy [1,27,30]. However, there has been disagreement whether simple appendectomy is sufficient to secure radicality for localized tumors or whether RSH is mandatory [5,6,8,9,16,20,31]. In addition, some studies suggest a prophylactic BSO in female patients, at the time of RSH due to the high propensity for ovarian metastases [5,6,9,16]. Two females of our cohort had metastases to the ovaries years after radical surgery for GCC and had a subsequent BSO. Based on our results we do not advocate for prophylactic BSO in female patients with localized GCC at diagnosis since evidence is lacking.

Some authors have suggested that localized GCCs <1 cm, without serosal, mesoappendiceal or caecal invasion, with free surgical margins and with a low proliferative index (less than 2 mitoses/10 HPF), should be treated with a simple appendectomy since metastases rarely develop in these patients [8,20,32]. Our results advocate for performing RSH in all GCC patients, as these patients had a significantly longer OS (89 months) vs. patients having only simple appendectomy (25 months). However, the latter group was small with risk of selection bias. Noteworthy is, the significant longer RFS of the radical treated subgroup of patients with localized GCC at diagnosis (16 months) compared to the radical treated group of patients with disseminated GCC (33 months), which may suggest that a true radicality is rarely obtained in disseminated cases.

In the presence of disseminated disease at time of diagnosis debulking surgery is recommended when possible followed by chemotherapy with regimens similar to colorectal adenocarcinomas [11]. A recent study advocates for cytoreductive surgery + HIPEC in GCC patients with peritoneal spread with an OS of 68 months [33]. The treatment strategy should be individually tailored taking potential side effects and complications into consideration. Age, lymph node involvement and dissemination plus the Tang Classification are some of the factors to be considered. Guidelines for choice of chemotherapy are lacking; the ENETS Guidelines 2012 advocate for first line treatment with 5-flourouracil-based combinations [1]. In agreement, we found a tendency towards longer survival for patients treated with colonic cancer regimes—however, the number of patients treated was low along with the risk of selection bias.

For GCCs a 5-year survival between 60%–85% has been reported [2,5,24], while one study observed only 45% 5-year survival, reflecting the great proportion of females with metastases included [8]. These data correspond to our results, showing a 5-year survival of 58% hence our cohort has a preponderance of females (67%) having 5-year survival of 49%. To have disseminated disease at diagnosis is a negative prognostic factor with a HR of 8. Further, we found that non-radical operation to be a negative prognostic factor with HR of 7, which reflects the subgroup with disseminated disease not having curative surgery. Focally positive staining pattern for CgA and negative/focally positive staining for synaptophysin were associated with poorer survival, probably due to a more poorly differentiation in these tumors. The obvious question must be whether localized and disseminated GCCs represent different disease entities?

## Conclusion

We found that the Tang classification and tumor staging are important prognostic factors along with negative/ focally positive synaptophysin in tissue specimen and non-radical surgery, while the Ki-67 index has no prognostic value.

In addition, we found that localized appendiceal GCCs occur equally in males and females, while disseminated GCCs are much more common in females indicating a more aggressive disease in women. Furthermore, the median age of patients with localized and disseminated disease was equal.

## Study type and ethical considerations

Data handling was handled in a non-personalized matter (patient-numbering). All patients had given informed consent to the treatment. The study is approved by the National Committee on Health Research Ethics.

## Supporting Information

S1 Table Stage Grouping*. * reference: TNM Classification of Malignant Tumours; UICC; 7th Edition; pp. 101–105.
(DOCX)

S2 Table Antibodies used in immunohistochemistry. (DOCX)

## References

[1] Pape UF, Perren A, Niederle B, Gross D, Gress T, et al (2012) ENETS Consensus Guidelines for the Management of Patients with Neuroendocrine Neoplasms from the Jejuno-Ileum and the Appendix Including Goblet Cell Carcinomas. Neuroendocrinology 95:135–156. 10.1159/000335629 22262080

[2] McCusker ME, Coté TR, Clegg LX, Sobin LH (2002) Primary malignant neoplasms of the appendix: a population-based study from the surveillance, epidemiology and end-results program, 1973–1998. Cancer 94: 3307–3312. 12115365 10.1002/cncr.10589

[3] Bosman FT, Carneiro F, Hruban RH (2010) WHO Classification of Tumours of the Digestive System International Agency for Research on Cancer.

[4] Subbuswamy SG, Gibbs NM, Ross CF, Morson BC (1974) Goblet cell carcinoid of the appendix. Cancer 34: 338–344. 4852178 10.1002/1097-0142(197408)34:2<338::aid-cncr2820340218>3.0.co;2-w

[5] Pahlavan PS, Kanthan R (2005) Goblet cell carcinoid of the appendix. World Journal of Surgical Oncology 3: 36 15967038 10.1186/1477-7819-3-36 PMC1182398

[6] Plöckinger U, Couvelard A, Falconi M, Sundin A, Salazar R, et al (2007) Consensus guidelines for the management of patients with digestive neuroendocrine tumours: well-differentiated tumour/carcinoma of the appendix and goblet cell carcinoma. Neuroendocrinology 87: 20–30. 17934252 10.1159/000109876

[7] Toumpanakis C, Standish RA, Baishnab E, Winslet MC, Caplin ME (2007) Goblet Cell Carcinoid Tumors (Adenocarcinoid) of the Appendix. Dis Colon Rectum 50: 315–322. 17195086 10.1007/s10350-006-0762-4

[8] Pham TH, Wolff B, Abraham SC, Drelichman E (2006) Surgical and chemotherapy treatment outcomes of goblet cell carcinoid: a tertiary cancer center experience. Ann Surg Oncol 13: 370–376. 16485156 10.1245/ASO.2006.02.016

[9] Tang LH, Shia J, Soslow RA, Dhall D, Wong WD, et al (2008) Pathologic Classification and Clinical Behavior of the Spectrum of Goblet Cell Carcinoid Tumors of the Appendix. The American Journal of Surgical Pathology 32: 1429–1443. 10.1097/PAS.0b013e31817f1816 18685490

[10] Landry CS, Woodall C, Scoggins CR, McMasters KM, Martin RCG (2008) Analysis of 900 Appendiceal Carcinoid Tumors for a Proposed Predictive Staging System. Arch Surg 143: 664–670. 10.1001/archsurg.143.7.664 18645109

[11] Holt N, Grønbæk H (2012) Goblet cell carcinoids of the appendix. The Scientific World Journal 2013: 543696–543696. 10.1155/2013/543696 23365545 PMC3556879

[12] Rindi G, Klöppel G, Couvelard A, Komminoth P, Körner M, et al (2007) TNM staging of midgut and hindgut (neuro) endocrine tumors: a consensus proposal including a grading system. Virchows Arch 451: 757–762. 17674042 10.1007/s00428-007-0452-1

[13] Sobin LH, Gospodarowicz MK, Wittekind C (2011) TNM Classification of Malignant Tumours. John Wiley & Sons.

[14] Mandai M, Konishi I, Tsuruta Y, Suginami N, Kusakari T, et al (2001) Krukenberg tumor from an occult appendiceal adenocarcinoid: a case report and review of the literature. Eur J Obstet Gynecol Reprod Biol 97: 90–95. 11435017 10.1016/s0301-2115(00)00503-0

[15] Hristov AC, Young RH, Vang R, Yemelyanova AV, Seidman JD, et al (2007) Ovarian metastases of appendiceal tumors with goblet cell carcinoidlike and signet ring cell patterns: a report of 30 cases. The American Journal of Surgical Pathology 31: 1502–1511. 17895750 10.1097/PAS.0b013e31804f7aa1

[16] Butler JA, Houshiar A, Lin F, Wilson SE (1994) Goblet cell carcinoid of the appendix. Am J Surg 168: 685–687. 7978019 10.1016/s0002-9610(05)80145-x

[17] Greene FL, Page DL, Fleming ID, Balch CM, Fritz AG (2002) Ajcc Cancer Staging Handbook Plus Eztnm. Springer.

[18] Van Eeden S, Offerhaus GJA, Hart AAM, Boerrigter L, Nederlof PM, et al (2007) Goblet cell carcinoid of the appendix: a specific type of carcinoma. Histopathology 51: 763–773. 18042066 10.1111/j.1365-2559.2007.02883.x

[19] Liu E, Telem DA, Warner RRP, Dikman A, Divino CM (2011) The role of Ki-67 in predicting biological behavior of goblet cell carcinoid tumor in appendix. The American Journal of Surgery 202:400–403. 21824598 10.1016/j.amjsurg.2010.08.036

[20] Bucher P, Gervaz P, Ris F, Oulhaci W, Egger JF, et al (2005) Surgical Treatment of Appendiceal Adenocarcinoid (Goblet Cell Carcinoid). World J Surg 29: 1436–1439. 16136284 10.1007/s00268-005-7958-y

[21] Grabowski P, Gribeta S, Arnold CN, Houmlrsch D, Goumlke RUD, et al (2005) Nuclear Survivin Is a Powerful Novel Prognostic Marker in Gastroenteropancreatic Neuroendocrine Tumor Disease. Neuroendocrinology 81: 1–9. 15809513 10.1159/000084892

[22] Koch CA, Vortmeyer AO, Diallo R, Poremba C, Giordano TJ, et al (2002) Survivin: a novel neuroendocrine marker for pheochromocytoma. Eur J Endocrinol 146: 381–388. 11888845 10.1530/eje.0.1460381

[23] Sukpan K, Settakorn J, Khunamornpong S, Cheewakriangkrai C, Srisomboon J, et al (2011) Expression of survivin, CD117, and C-erbB-2 in neuroendocrine carcinoma of the uterine cervix. Int J Gynecol Cancer 21: 911–917. 10.1097/IGC.0b013e31821a2567 21633298

[24] Kanthan R, Saxena A, Kanthan SC (2001) Goblet cell carcinoids of the appendix: immunophenotype and ultrastructural study. Arch Pathol 125: 386–390. 11231488 10.5858/2001-125-0386-GCCOTA

[25] Ramnani DM, Wistuba I, Behrens C, Gazdar AF (2000) K-ras and p53 mutations in the pathogenesis of classical and goblet cell carcinoids of the appendix Cancer. Wiley Online Library 10.1002/(sici)1097-0142(19990701)86:1<14::aid-cncr4>3.0.co;2-x 10391558

[26] Roy P, Chetty R (2010) Goblet cell carcinoid tumors of the appendix: An overview. World J Gastrointest Oncol 2: 251–258. 10.4251/wjgo.v2.i6.251 21160637 PMC2998842

[27] Janson ET, Sorbye H, Welin S, Federspiel B, Grønbaek H, et al (2014) Nordic guidelines 2014 for diagnosis and treatment of gastroenteropancreatic neuroendocrine neoplasms. Acta Oncol 53: 1284–1297. 10.3109/0284186X.2014.941999 25140861

[28] Lee KS, Tang LH, Shia J, Paty PB, Weiser MR, et al (2013) Goblet cell carcinoid neoplasm of the appendix: Clinical and CT features. Eur J Radiol 82: 85–89. 10.1016/j.ejrad.2012.05.038 23088880

[29] Binderup T, Knigge U, Loft A, Mortensen J, Pfeifer A, et al (2010) Functional Imaging of Neuroendocrine Tumors: A Head-to-Head Comparison of Somatostatin Receptor Scintigraphy, 123I-MIBG Scintigraphy, and 18F-FDG PET. Journal of Nuclear Medicine 51: 704–712. 10.2967/jnumed.109.069765 20395333

[30] Boudreaux JP, Klimstra DS, Hassan MM, Woltering EA, Jensen RT, et al (2010) The NANETS consensus guideline for the diagnosis and management of neuroendocrine tumors: well-differentiated neuroendocrine tumors of the Jejunum, Ileum, Appendix, and Cecum 39: 753–766. 10.1097/MPA.0b013e3181ebb2a5 20664473

[31] Varisco B, McAlvin B, Dias J, Franga D (2004) Adenocarcinoid of the appendix: is right hemicolectomy necessary? A meta-analysis of retrospective chart reviews. Am Surg 70: 593–599. 15279181

[32] Subbuswamy SG, Gibbs NM, Ross CF, Morson BC (1974) Goblet cell carcinoid of the appendix. Cancer 34: 338–344. 4852178 10.1002/1097-0142(197408)34:2<338::aid-cncr2820340218>3.0.co;2-w

[33] McConnell YJ, Mack LA, Gui X, Carr NJ, Sideris L, et al (2014) Cytoreductive Surgery with Hyperthermic Intraperitoneal Chemotherapy: An Emerging Treatment Option for Advanced Goblet Cell Tumors of the Appendix. Ann Surg Oncol 21: 1975–1982. 10.1245/s10434-013-3469-5 24398544

