# Supplementary material for: Goblet Cell Carcinoids: Characteristics of a Danish Cohort of 83 Patients
Source: PLoS One. 2015 Feb 11;10(2):e0117627. doi: 10.1371/journal.pone.0117627 (PMC4324995; doi:10.1371/journal.pone.0117627)
Supplement: S1 Table — * reference: TNM Classification of Malignant Tumours; UICC; 7th Edition; pp. 101–105. (DOCX) [file pone.0117627.s001.docx]

**Supporting Information**

**Table S1. Stage Grouping***

| **Table S1. Stage Grouping*** | |  |  |
| --- | --- | --- | --- |
| Stage 0 | Tis | N0 | M0 |
| Stage I | T1, T2 | N0 | M0 |
| Stage II | T3, T4 | N0 | M0 |
| Stage III | Any T | N1,N2 | M0 |
| Stage IVA | Any T | Any N | M1a |
| Stage IVB | Any T | Any N | M1b |
| ** reference: TNM Classification of Malignant Tumours; UICC; 7th Edition; pp. 101-105* | | | |
