# Supplementary material for: Goblet Cell Carcinoids: Characteristics of a Danish Cohort of 83 Patients
Source: PLoS One. 2015 Feb 11;10(2):e0117627. doi: 10.1371/journal.pone.0117627 (PMC4324995; doi:10.1371/journal.pone.0117627)
Supplement: S2 Table — (DOCX) [file pone.0117627.s002.docx]

**Supporting Information**

**Table S2. Antibodies used in Immunohistochemistry**

| **Table S2. Antibodies used in immunohistochemistry** | | |  |  |
| --- | --- | --- | --- | --- |
| **ANTIBODY** | **CLONE /CODE** | **HOST** | **PROVIDER** | **DILUTION** |
| Chromogranin A (CgA) | A0430 | Polyclonal Rabbit | Dako Denmark A/S, Glostrup, Denmark | 1:2000 |
| Synaptophysin | Svp 88 | Monoclonal Mouse | Novestra Ltd, Newcastle, UK | 1:50 |
| Serotonin | 5H7-H209 | Monoclonal Mouse | Dako Denmark A/S, Glostrup, Denmark | 1:20 |
| Ki67 | MIB1 | Monoclonal Mouse | Dako Denmark A/S, Glostrup, Denmark | 1:100 |
| p53 | D0-7 | Monoclonal Mouse | Dako Denmark A/S, Glostrup, Denmark | 1:200 |
| MUC1 | E29 | Monoclonal Mouse | Dako Denmark A/S, Glostrup, Denmark | 1:50 |
| MUC2 | CCP58 | Monoclonal Mouse | Dako Denmark A/S, Glostrup, Denmark | 1:150 |
| Survivin | 2463-1 | Monoclonal Rabbit | Epitomics, Burlingame, USA | 1:100 |
| *Formalin-fixed paraffin-embedded tissue samples were cut at 4 μm thick sections and mounted on coated slides (Dako Flex IHC microscope slides™, Glostrup, Denmark). To remove the paraffin EZ-prep from Ventana© (Tucson, USA) was used. Afterwards slides were pretreated with Ventana CC1™ (cell conditioning pH8.5) for 64 min. and incubated with antibody 32 min./36°C diluted 1:100 in Dako antibodydiluent S2022™ in Ventana Benchmark Ultra™. The reaction was visualized by using Ventana Ultra View DAB-kit™. Afterwards the sections were counterstained with Ventana Haematoxylin™ for 8 min.* | | | | |
